# Local and Regional Effects on Community Structure of Dung Beetles in a Mainland-Island Scenario

**DOI:** 10.1371/journal.pone.0111883

**Published:** 2014-10-30

**Authors:** Pedro Giovâni da Silva, Malva Isabel Medina Hernández

**Affiliations:** Programa de Pós-Graduação em Ecologia, Departamento de Ecologia e Zoologia, Universidade Federal de Santa Catarina, Florianópolis, Santa Catarina, Brazil; University of Waikato (National Institute of Water and Atmospheric Research), New Zealand

## Abstract

Understanding the ecological mechanisms driving beta diversity is a major goal of community ecology. Metacommunity theory brings new ways of thinking about the structure of local communities, including processes occurring at different spatial scales. In addition to new theories, new methods have been developed which allow the partitioning of individual and shared contributions of environmental and spatial effects, as well as identification of species and sites that have importance in the generation of beta diversity along ecological gradients. We analyzed the spatial distribution of dung beetle communities in areas of Atlantic Forest in a mainland-island scenario in southern Brazil, with the objective of identifying the mechanisms driving composition, abundance and biomass at three spatial scales (mainland-island, areas and sites). We sampled 20 sites across four large areas, two on the mainland and two on the island. The distribution of our sampling sites was hierarchical and areas are isolated. We used standardized protocols to assess environmental heterogeneity and sample dung beetles. We used spatial eigenfunctions analysis to generate the spatial patterns of sampling points. Environmental heterogeneity showed strong variation among sites and a mild increase with increasing spatial scale. The analysis of diversity partitioning showed an increase in beta diversity with increasing spatial scale. Variation partitioning based on environmental and spatial variables suggests that environmental heterogeneity is the most important driver of beta diversity at the local scale. The spatial effects were significant only at larger spatial scales. Our study presents a case where environmental heterogeneity seems to be the main factor structuring communities at smaller scales, while spatial effects are more important at larger scales. The increase in beta diversity that occurs at larger scales seems to be the result of limitation in species dispersal ability due to habitat fragmentation and the presence of geographical barriers.

## Introduction

Community ecology aims to understand and explain the processes that influence the patterns of distribution, abundance and composition of species [1], [2] over space and time [3], both locally and regionally [4], [5]. Community structure may be influenced by several ecological processes that involve biotic and abiotic factors operating at different spatial and temporal scales [6]–[8]. When we consider large scales, historical, evolutionary and stochastic processes become critical to the understanding of these patterns [5], [9]–[11]. Further, studying only local processes may not be sufficient for understanding how communities are structured locally and regionally [5], because local and regional processes may act in different ways in relation to the increase or decrease in species diversity [11].

Despite the large number of mechanisms (theories and models) proposed as drivers of patterns of species distribution, only four processes are fundamentally involved: selection, drift, speciation and dispersal [2]. Three main hypotheses are proposed to explain the origin of beta diversity (i.e., variation in the identities and relative abundance of species among sites) with respect to these processes [12]. The first hypothesis suggests that the species composition may be stable over large areas, and that biological interactions (e.g., competition inter- and/or intraspecific) play an important role in maintaining beta diversity [12]. The second hypothesis states that species composition varies in a random and autocorrelated way, emphasizing spatially limited dispersal [12]. The last hypothesis suggests that species distribution is driven by environmental conditions, and that landscapes are mosaics in which local environmental drivers control species composition [12]. These hypotheses seem to be somewhat related regardless of the organismal group or ecosystem, and testing them is crucial for elucidating issues on ecosystem functioning and biodiversity conservation initiatives [12].

In community ecology there exists a variety of concepts and methodologies commonly employed by ecologists to measure beta diversity and to identify the processes related to its generation [12]–[17]. Recent approaches have been based on the dependence of environmental, spatial and random processes, with the goal of explaining which processes have more influence on beta diversity, e.g., by using variation partitioning methods [18] on composition or abundance community data among groups of explanatory variables (e.g., environmental and spatial) [12]. These methods are used to attempt to explain how beta diversity is influenced by environmental and/or spatial factors, or by random factors [19]. Despite being criticized [20], [21], variation partitioning has long been used in the context of metacommunity theory [1] and it highlights the importance of increasing the spatial scale in understanding the ecological processes structuring biological communities locally and regionally [22].

A metacommunity is defined as a set of communities connected by the dispersal of multiple interacting species [1], [22]. There are four theoretical paradigms (models) to explain metacommunity dynamics (species sorting, mass effects, patch dynamics and neutral) and they take into account three (drift, selection and dispersal) of the four basic processes aforementioned [2]; differences in species dispersal ability and environmental characteristics are important factors for determining which model best describes the metacommunity [1], [22]. Mass effects (high dispersal) and patch dynamics (low dispersal) would be variations of species sorting (efficient dispersal), as there are different levels of dispersal ability of species in each metacommunity model [23]. The adoption of metacommunity theory has led to substantial changes in the way that ecologists interpret ecological phenomena at both local and metacommunity (regional) scales [1].

A key point in assessing the relative importance of proposed metacommunity processes is the identification and use of environmental and/or spatial gradients as study scenarios [12]. Direct gradient ordination techniques (e.g., redundancy analysis) followed by variation partitioning [18] allows determination of the fraction of beta diversity explained solely by environmental or spatial predictors, and by shared effects of both sets of predictors [12]. The prevalence of environmental effects indicates species sorting, the predominance of spatial effects indicates neutral processes, historical events and/or dispersal limitation, and shared effects of both environmental and spatial predictors indicate species sorting, dispersal limitation or a combination of both (mass effects and patch dynamics sensu [1]) [23], [24]. The relative importance of metacommunity paradigms is still dependent on spatial scale, spatial extent or spatial distances between sites [25], [26], and varies between environments and groups of species due to inherent differences of ecosystem type and species dispersal ability [27]. Recent techniques have also allowed the identification of species and sites that may contribute to beta diversity along an ecological gradient by using community dataset total variance as an estimate of beta diversity [15].

The Brazilian Atlantic Forest is one the most threatened biomes in terms of biodiversity conservation [28]. About 12% of its original size, it is highly fragmented with a high degree of isolation, existing primarily in intermediate successional state [29]. Less than 2% of Atlantic Forest areas are located in protected zones [29], despite being considered global biodiversity hotspots [30]. Historically, the coast of Brazil has always showed the highest population and industrial concentration, and thus, the Atlantic Forest has been affected by the growth and development of the country [29]. An understanding of how species respond to anthropogenic modifications to the structure or complexity of habitats is fundamental for the development of future conservation initiatives, especially for organisms that play key roles in the maintenance and/or restoration of ecosystems, such as dung beetles (Coleoptera: Scarabaeidae: Scarabaeinae).

Dung beetles feed on decaying organic matter (e.g., mammalian feces, animal carcasses, rotting vegetation) [31] and they play several ecosystem services [32]. In tropical ecosystems they are used as indicators of diversity, as well as for monitoring environmental changes, because they respond quickly in terms of species composition, richness, abundance and biomass to the effects caused by habitat destruction, fragmentation and/or isolation [33]–[37]. These beetles are easily sampled using standardized, efficient and low-cost sampling methods [34]. They have wide distribution and are correlated with other taxa (e.g., mammals) [33], [34], [38]. Therefore, community changes have potential to affect ecological functions performed by dung beetles and hence, ecosystem function [35], [39]–[42]. As such, dung beetles are an excellent model system [31] with which to investigate the main processes that influence community structure in Atlantic Forest regions.

Therefore, the aim of our study was to investigate the effect of spatial scales on the patterns of species diversity of dung beetles in Brazilian Atlantic Forest and to identify the mechanisms that drive these patterns applying aspects of metacommunity theory. We tested the hypothesis that the distribution of dung beetles in the Atlantic Forest is associated with differences in forest structure and that high levels of beta diversity will be found with increasing spatial scale due to dispersal limitation. Our predictions are as follows: (i) because dung beetles are sensitive to environmental changes, environmental gradients should result in high beta diversity among sites via species sorting, (ii) due to differences in habitat structure of each site, environmental characteristics and dung beetle species distribution are spatially structured, (iii) because of dispersal limitation among areas (mainland-island and fragmented landscape), the spatial effect has high importance in structuring communities at increased spatial scales.

## Materials and Methods

### Study area

**Figure 1:**
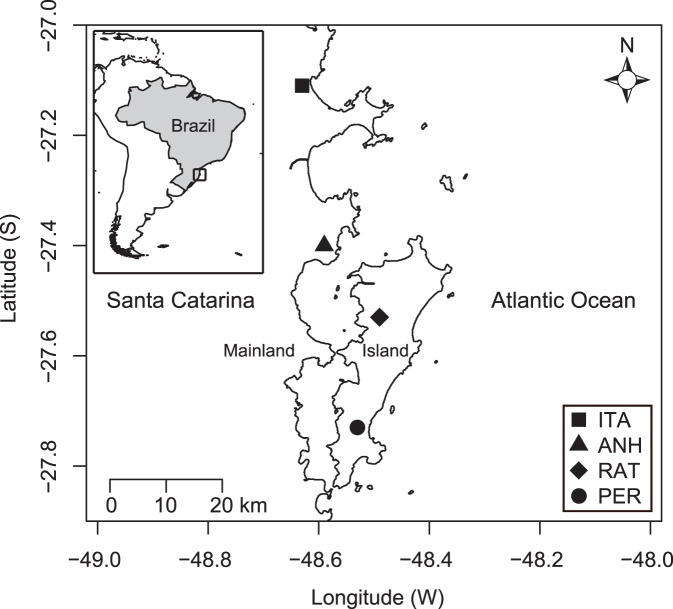
Map of the study region. Location of the four areas sampled in eastern Santa Catarina state, Brazil. ANH: Environmental Protection Area of Anhatomirim; ITA: Permanent Protection Area of Itapema; PER: Lagoa do Peri Municipal Park; RAT: Permanent Protection Area of Ratones.

The study was conducted at four large Atlantic Forest areas in the state of Santa Catarina, Brazil, two on the island of Santa Catarina (Florianópolis city) and two on the mainland, both on the east coast (Figure 1). The island of Santa Catarina is approximately 54 km north-south and maximally 18 km wide, with a total land area of 424.4 km^2^. The distance between the mainland and the island varies greatly, with minimum of 500 m and maximum around 10 km.

On the island, the study areas were within the Lagoa do Peri Municipal Park (PER, 27°43′30″S, 48°32′18″W) and the Permanent Protection Area of Ratones (RAT, 27°31′52″S, 48°30′45″W). On the mainland, the areas sampled were within the Environmental Protection Area of Anhatomirim in Governador Celso Ramos city (ANH, 27°25′1″S, 48°34′25″W), and in Permanent Protection Area in Itapema city (ITA, 27°05′13″S, 48°35′54″W). According to the Brazilian Forest Code (Law n°. 12.651/2012), permanent protection areas are sites with characteristics that have the environmental function of preserving water, biodiversity resources, and landscape and geological stability, and for facilitation floral and faunal gene flow. The distance among areas is approximately 21 km between PER and RAT, 34 km between PER and ANH, 71 km between PER and ITA, 13.5 km between ANH and RAT, 50 km between ITA and RAT, and 37 km between ANH and ITA. The altitude of the sampling sites ranged between 28 and 265 m. All sites sampled are near the Brazilian Atlantic coastline and have dense rain forest vegetation within the Atlantic Forest biome, with various levels of vegetation succession [43]. According Köeppen classification, the climate in the eastern region of Santa Catarina is Cfa, humid subtropical (mesothermal) without dry season, with hot summers (average of 25°C) and well distributed rainfall throughout the year, with annual average of 1500 mm approximately [43]. Santa Catarina shows four seasons of the year well defined.

### Scarabaeinae sampling

We sampled dung beetles using baited pitfall traps, as they are a highly efficient method to capture this group [44]. The traps were made with plastic containers (15 cm diameter×20 cm depth) buried with the top edge at ground level, allowing insects to fall in. To prevent overflow, the traps were protected against rain using a small sheet supported by wooden sticks, placed approximately 10 cm above the trap. A mixture (300 ml) of water and detergent (neutral) was added to each container to catch and kill insects. We used human feces and rotting flesh (aged in plastic container at room temperature three days prior to sampling) as bait to attract dung beetles, as both satisfy the two main eating habits of dung beetles – coprophagy and necrophagy, respectively [31]. Approximately 30 g of both baits were wrapped in thin cloth and tied in the central part of the rain protection above the traps.

The insects collected were sorted, mounted on entomological pins and dried in an oven (60°C for 72 h), then weighed on a precision balance (0.0001 g). Species identities were confirmed by experts. The beetles were deposited in the Entomological Collection of the Centro de Ciências Biológicas at the Universidade Federal de Santa Catarina, Brazil. We thank the Instituto Chico Mendes de Conservação da Biodiversidade (ICMBio/MMA) and Fundação do Meio Ambiente (FATMA-SC) for permission to collect (permit #32333-3 to MIMH). The field study did not involve endangered or protected species. Dataset S1 provides the database of values for abundance and biomass of dung beetle species across the study sites.

### Sampling design

Samples were taken at five different forested (hillside) sites within each sampling area. Each site contained five pairs of traps spaced 5–10 m apart, each pair containing the two kinds of bait. The pairs were spaced 50 m apart, as a minimum distance of 50 m decreases the influence between sets of traps in sampling Scarabaeinae [45]. Each pair of traps was regarded as a sampling point, and remained in the field for 48 h prior to collection.

The samplings were carried out during the summer of 2012 (January and February), because of high temperatures, and it being the period of greatest dung beetle abundance in subtropical regions in Brazil [46], [47]. Due to the spatial configuration of our sampling design, the large distance among the four areas, and the effect of spatial discontinuity between mainland-island, the sampling sites showed a hierarchical distribution. Thus, it was possible to investigate the variation in dung beetle communities at three spatial scales, i.e. mainland-island, areas, and sites. Sites represent the local spatial scale, i.e., the smallest spatial extent in our study that encompasses five sampling points. Areas represent the intermediate spatial scale with five sites per area. Mainland-island represents the regional spatial scale, i.e., the largest spatial extent in our study that encompasses two areas each one. Variation in dung beetle species composition, number of individuals, and dry biomass was used to assess the influence of environmental and spatial factors at each spatial scale.

### Environmental variables

We measured 20 environmental variables related to habitat structure to test their influence on dung beetle distribution. Differences in environmental conditions (environmental variables measured) among sampling sites is defined as environmental heterogeneity. Measurement was performed using the adapted point-centered quarter method [48], [49]. This method was chosen for its simplicity and common use in phytosociological surveys [50]. Briefly, a plastic pipes crossing in an x-shape were placed in the center of each pair of traps (i.e., at each sampling point), dividing the sampling point into four quadrants (northwest, southwest, southeast and northeast). Tree, shrub and soil environmental variables were measured in each quadrant as follows: (1) circumference at breast height when diameter at breast height >5 cm), (2) height, (3) top diameter and (4) distance away from the nearest tree to the center of cross, (5–8) same measures for the greater tree distant up to 10 m, (9–12) similar measures for shrubs (circumference at ankle height when <5 cm and with a minimum height of 1 m), (13) land slope, (14) canopy cover, (15) percentage of leaf litter cover, (16) green cover and (17) exposed soil, (18) height and (19) dry biomass of leaf litter, and (20) altitude.

The height of trees and shrubs was visually estimated with a ruler of 4 m length. Circumference and distance were measured with a millimeter tape measure. The percentage of litter, green cover, and exposed soil coverage in each quadrant was estimated in different classes (0–5, 6–25, 26–50, 51–75, 76–95, 96–100%) using a square of 1 m plastic pipes, placed about 20 cm away from the cross. Land slope was obtained at the center of the square using an inclinometer. Litter height was measured using a mm ruler at five points inside the square (near each corner and in the center). A five-inch square was constructed in the center of the 1 m square, and a portion of litter was removed. Litter was later dried in an oven (60°C for 72 hours) and weighed to obtain dry biomass. Using the same classes described above, the percentage of canopy cover was visually estimated using a hollow square of 10 cm side length, placed at a distance of 60 cm from the eye of the observer at a 20° angle in relation to the zenith [50]. Altitude was obtained using a hand-held GPS at ground level. The basal area of trees and shrubs was calculated from the trunk circumference (based on the area of the circle). For each variable, a measure of central tendency was calculated based on the Shapiro-Wilk normality test. Thus, each environmental variable represented a central value (mean or median, as appropriate) of the four measures of each point; this was done to minimize the effects of visual estimation. A subset of the variables analyzed (three basal area, three heights, DBH) is used by the Conselho Nacional do Meio Ambiente, the Brazilian Council of Environmental issues, to characterize successional stages of Atlantic Forest in the state of Santa Catarina [51]. Dataset S1 provides the database of values for each environmental variable across the study sites.

### Spatial variables

Spatial predictors were created using a method called Principal Coordinates of Neighbour Matrices [19], which is part of a set of spatial eigenfunction analyses called Moran’s Eigenvector Maps [52]. The creation of spatial predictors was performed using *create.MEM.model* function [25] for the program R [53], because the sampling sites in our study showed a spatial hierarchical structure [54] with large distances between sites in different areas. This function produces a staggered matrix arranged in blocks from the geographical coordinates, generating information on the number of blocks (or groups) and sampling sites in each block [54]. Each block represents the hierarchical spatial distribution of sampling points, and in the staggered matrix the blocks from another hierarchy receive value of zero (0) for each spatial variable created. These variables represent the spatial variation at different spatial scales and may be used as predictors in gradient analysis to model the spatial relationship of the community data [25]. To create the spatial variables, we used data from geographic coordinates (Universal Transverse Mercator) obtained at each sampling point using a hand-held GPS. Dataset S1 provides the database of geographic coordinates for each study site.

### Data analysis

#### Beta diversity across spatial scales

A recent approach called “true diversity” [55] has been used to partition diversity into its different components in an additive or multiplicative way [14]. The additive partitioning approach (γ = α+β1+β2+β3) was used to estimate the beta diversity at the different spatial scales. Alpha (α) is the average species richness in local communities, while gamma (γ) refers to the total richness observed in the entire set of samples. Each component of beta diversity refers to different spatial scales studied: β1 = between sampling sites, β2 = between areas, β3 = between mainland-island. We used data on species richness and individual abundance (i.e., true Shannon diversity) for the hierarchical analysis of diversity partitioning. Partitioned components of diversity based on abundance were natural log-transformed to make them additive (i.e., Shannon entropy [55]). These analyses were performed in Partition 3.0 program [56].

#### Species and local contributions to beta diversity

The total beta diversity (BD_Total_) was analyzed by calculating the total variance of the species matrix using *beta.div* function [15] for R program [53]. This method calculates the total sum of squares of the species matrix and from it, one may obtain an index of the total data variance that represents the total beta diversity, and it may be compared among sampling units. The BD_Total_ may then be partitioned in Species Contribution to Beta Diversity (SCBD, or degree of variation of the species along the studied area) and Local Contribution to Beta Diversity (LCBD, or comparative indicators of ecological uniqueness of the sampling sites) [15]. The values of LCBD were tested using random and independent permutations (in columns) of the species matrix, testing whether species are randomly and independently distributed between sampling sites [15]. This approach was used to identify the species and sites that contributed most to the beta diversity index throughout the spatial gradient. Before running the analysis, species data (composition, abundance and biomass) were Hellinger-transformed, after which Euclidean distance was used in the execution of the analysis. We used Spearman correlation to assess the association between the values of LCBD and species richness, abundance and biomass, in order to determine whether sampling site contribution is related to the number of species, number of individuals, and total biomass. Analyses were performed in R 3.0.1 program [53].

#### Variation partitioning explained by explanatory variables

Double stopping criterion [57] was used as forward selection procedure of explanatory variables in order to avoid type I error, and to avoid overestimating the amount of explained variance in the species matrix before variation partitioning [54], [57]. Variation partitioning allows partitioning the variation in the species data explained by pure environmental effects [a], spatially structured environmental variables [b], pure spatial effects [c], and unexplained variation (i.e., residuals or fraction [d]) [18], [58]. This method estimates and tests the percentage of variation (R^2^_adj_) attributed to each unique set of explanatory variables [18]. Three steps were necessary to perform the variation partitioning: (1) implementation of a redundancy analysis (RDA) with sets of environmental and spatial variables, (2) a second RDA with environmental data, controlling for spatial effects (E | S), (3) a third RDA with spatial data, controlling for environmental effects (S | E) [18]. Variation partitioning was performed for the composition, abundance and biomass of dung beetles at each spatial scale studied. Before running RDAs, species datasets were Hellinger-transformed in order to eliminate the disparity between values [59]. The proportion of variance explained by each set of explanatory variables is described by R^2^_adj_ according to the Ezekiel correction [18], and significance levels are calculated by permutation tests (N = 999) [54]. We tested for a linear spatial trend and found a significant longitudinal trend for dung beetle composition data (F = 3.34; df = 2; p<0.01), abundance (F = 6.77; df = 2; p<0.01), and biomass (F = 7.35; df = 2; p<0.01). Thus, all datasets were detrended prior to the analyses [54]. R^2^_adj_ values were indicated in percentage format in the text and tables. The analyses were conducted in R 3.0.1 program [53] using Packfor [60] and Vegan [61] packages.

## Results

### Species richness, abundance and biomass across spatial scales

Regionally, we collected a total of 3,004 individuals of Scarabaeinae, belonging to 21 species (Table S1). The mainland and the island had the same total number of species (16), sharing 11. On the island, the number of individuals was 2.5 higher, and total biomass was 2 times higher compared to the mainland. Among areas, RAT had the greatest number of species and individuals, and greatest biomass, followed by PER (both island areas). Only eight species (38.1%) were shared by all four areas.

The number of species per site ranged between five and 14 (Table S1). Only one species occurred in all sampling sites (*Canthon rutilans cyanescens*). Three species were sampled at least 19 sites (*Deltochilum morbillosum*, *Deltochilum multicolor*, and *Dichotomius sericeus*). Five species were responsible for 92.8% of the total dung beetle biomass (*D. sericeus*, *Coprophanaeus saphirinus*, *C. rutilans cyanescens*, *D. multicolor* and *D. morbillosum*) (see Figure S1 for a spatial comparison of species richness, abundance and biomass).

### Beta diversity across spatial scales

**Figure 2:**
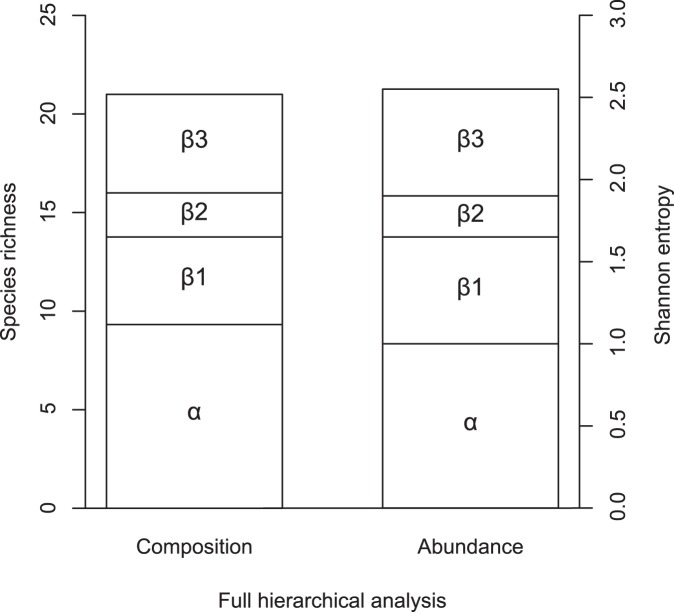
Full hierarchical analysis of diversity partitioning. The partitioning was performed for species richness and Shannon entropy of dung beetles. α = local diversity, β1 = diversity among sites, β2 = diversity among areas, β3 = diversity among mainland-island.

The hierarchical partitioning analysis of diversity based on species richness data showed a large contribution of regional (β3 = 5 species) and local (β1 = 4.4 species) spatial scales to gamma diversity (Figure 2). Beta diversity among areas (β2 = 2.2 species) was relatively small when compared to other spatial scales. A similar pattern was observed for Shannon entropy based on species abundance. The hierarchical partitioning of diversity analysis also indicated a small contribution of β2, and a large relative contribution of β3 and β1, respectively.

Our results show that there were five species found only on the mainland (*Bdelyrus braziliensis*, *Coprophanaeus dardanus*, *Deltochilum furcatum*, *Dichotomius quadrinodosus*, and *Eurysternus cyanescens*), and five only found on the island (*Dichotomius* sp., *Eurysternus parallelus*, *Paracanthon* aff. *rosinae*, *Uroxys* sp. 1, and *Uroxys* sp. 2). *Bdelyrus braziliensis* and *Eurysternus cyanescens* were found only in ANH, on the mainland. *Coprophanaeus dardanus*, *Deltochilum furcatum* and *Dichotomius quadrinodosus* occurred only in ITA, on the mainland. *Dichotomius* sp. and *Uroxys* sp. 2 occurred only in RAT, on the island. *Paracanthon* aff. *rosinae* and *Uroxys* sp. 1 were only shared between RAT and PER.

### Species and local contributions to beta diversity index

**Figure 3:**
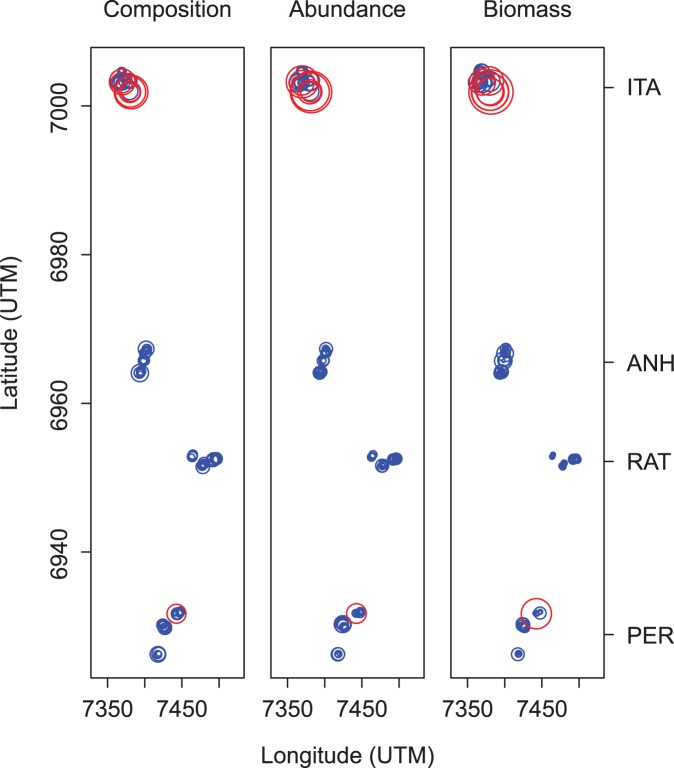
Map of the sampling points showing significant values (red) of the local contribution to beta diversity (LCBD). LCBD analysis used composition, abundance and dry biomass data. ANH: Environmental Protection Area of Anhatomirim; ITA: Permanent Protection Area of Itapema; PER: Lagoa do Peri Municipal Park; RAT: Permanent Protection Area of Ratones. The circles are proportional to the total value of LCBD for each analysis.

The partitioning of the total variance in components of the contribution of species and sites to beta diversity showed different results when data on composition, abundance and biomass of dung beetles were analyzed. For composition, the total sum of squares (SS_Total_) was 38.183 and the index of variance of beta diversity (BD_Total_) was 0.395 for dung beetle data across all sampling sites. SCBD values ranged between 0.002 and 0.145, and 10 species contributed above the mean (0.047) to beta diversity (Table 1 left). The values of LCBD ranged between 0.003 and 0.032, indicating the uniqueness of the dung beetle community at each sampling site. Six sampling points were statistically significant to beta diversity (Figure 3A), all within two ITA sites and one PER site. LCBD values were negatively correlated with species richness (r = −0.45, p<0.001) indicating that, in general, sites with unique species composition have a low number of species.

**Table 1:** Partitioning of the total variance in species contribution to beta diversity (SCBD) based on the beta diversity index (BD_Total_) and the total sum of squares (SS_Total_).

Species	Composition	Abundance	Biomass
	SS_Total_ = 38.183	SS_Total_ = 35.691	SS_Total_ = 35.275
	BD_Total_ = 0.395	BD_Total_ = 0.360	BD_Total_ = 0.356
*Canthidium* aff. *trinodosum*	0.121	0.141	
*Canthon luctuosus*	0.061		
*Canthon rutilans cyanescens*	0.055	0.153	0.113
*Coprophanaeus saphirinus*	0.100	0.123	0.235
*Deltochilum multicolor*	0.145	0.116	0.155
*Deltochilum morbillosum*	0.109	0.101	0.067
*Deltochilum rubripenne*	0.068		
*Dichotomius sericeus*	0.059		0.230
*Phanaeus splendidulus*	0.063		0.49
*Uroxys* sp. 1	0.053		

For abundance data, the SS_Total_ was 35.691 and the BD_Total_ was 0.360. SCBD values ranged between 0.0003 and 0.179, and five species contributed above the mean (0.047) to beta diversity (Table 1 center). LCBD values ranged between 0.002 and 0.040, and seven sampling points were statistically significant (Figure 3B), all occurring in the same two ITA sites sampled for composition data. LCBD values were negatively correlated with abundance at each sampling point (r = −0.32, p<0.001), demonstrating that sites with unique species composition, in general, have low abundance.

For biomass data, the SS_Total_ was 35.275 and the BD_Total_ was 0.356. SCBD ranged between 6.915e^−06^ and 0.235, and six species contributed above the mean (0.047) to beta diversity (Table 1 right). LCBD values ranged between 0.002 and 0.042, and eight sampling points were statistically significant (Figure 3C). LCBD values were negatively correlated with biomass of each sampling point (r = −0.49, p<0.001), and as well as to species richness and abundance, suggesting that sites with unique species composition, in general, have low dung beetle biomass.

Only four species (*C. rutilans cyanescens*, *C. saphirinus*, *D. multicolor* and *D. morbillosum*) contributed to the beta diversity index taking into account the composition, abundance and biomass of dung beetles. Although these species are very common among the sampled sites, this result suggests that they had strong local spatial variation in terms of occurrence, number of individuals and total biomass between sites.

### Environmental and spatial effects on community variation at different spatial scales

The variation partitioning based on community composition, abundance and biomass showed different responses at each spatial scale when we analyzed each species dataset. The variation in species composition at mainland-island scale showed a greater and significant environmental effect (Table 2A left). Altitude was the only environmental variable selected to compose the environmental model and it explained 4.5% of variation at this scale. When the spatial configuration was removed from the environmental model the explanation decreased to 4.4%. The spatial effect was not significant for variation in species composition, and after the environmental effect was removed the spatial model was still not significant. The variation explained by spatially structured environmental variables [b] explained only 0.02%.

**Table 2:** Partitioning of variation in dung beetle communities at three spatial scales using redundancy analysis on composition, abundance and biomass.

		Composition				Abundance				Biomass			
		R^2^_adj_	DF	F	P	R^2^_adj_	DF	F	P	R^2^_adj_	DF	F	P
A) Mainland-island													
E	[a + b]	**4.5** a	1	5.62	0.001	**9.9** ^e^	3	4.62	0.001	**9.9** ^e^	3	4.61	0.001
S	[b + c]	0.4^b^	2	1.19	0.268	**1.4** ^f^	1	2.40	0.016	**2.8** ^b^	1	3.88	0.003
E | S	[a]	**4.4**	1	5.53	0.001	**10.0**	3	4.70	0.001	**9.9**	3	4.69	0.001
S | E	[c]	0.4	2	1.19	0.242	**1.6**	1	2.68	0.006	**2.8**	1	4.11	0.001
													
B) Areas													
E	[a + b]	**4.5** a	1	5.62	0.001	**9.9** ^e^	3	4.62	0.001	**9.9** ^e^	3	4.61	0.001
S	[b + c]	**13.2** ^c^	5	4.02	0.001	**17.4** ^g^	7	3.98	0.001	**16.6** ^h^	6	4.28	0.001
E | S	[a]	**1.2**	1	2.27	0.012	**7.3**	3	3.97	0.001	**8.4**	3	4.45	0.001
S | E	[c]	**9.9**	5	3.27	0.001	14.8	7	3.69	0.001	**15.1**	6	4.21	0.001
													
C) Sites													
E	[a + b]	**4.5** a	1	5.62	0.001	**9.9** ^e^	3	4.62	0.001	**9.9** ^e^	3	4.61	0.001
S	[b + c]	−11.2^d^	40	0.75	0.999	−20.2^d^	40	0.58	1.000	−22.9^d^	40	0.53	1.000
E | S	[a]	**9.0**	1	6.19	0.001	**15.8**	3	3.98	0.001	**17.2**	3	4.20	0.001
S | E	[c]	−6.7	40	0.83	0.976	−14.2	40	0.67	0.999	−15.5	40	0.64	0.999

E: environmental model, S: spatial model, constructed from MEM variables, E | S: environmental model without spatial patterns within each spatial scale, S | E: spatial model without environmental patterns within each spatial scale, R^2^_adj_: data variation explained by the model (values are in percentage), DF: degrees of freedom of model. Significant models are in bold.

a Environmental model constructed from the altitude variable; ^b^Spatial model constructed from the MEM1 and MEM2 variables; ^c^Spatial model constructed from the MEM4, MEM9, MEM5, MEM3, and MEM1 variables; ^d^Spatial model constructed from all MEM variables; ^e^Environmental model constructed from the altitude, green cover and land slope variables; ^f^Spatial model constructed from the MEM1 variable; ^g^Spatial model constructed from the MEM4, MEM9, MEM3, MEM7, MEM1, MEM2, and MEM5 variables; ^h^constructed from the MEM4, MEM3, MEM2, MEM5, MEM7, and MEM1 variables.

At the scale of areas, spatial effects were stronger than environmental effects, and it explained 13.2% of the variation in species composition (Table 2B left). After environmental effects were removed, the spatial model explained 9.9% of the variation in the data. The environmental model, which was composed of altitude only, explained only 1.2% after spatial effects were removed. Spatially structured environmental variables [b] explained 3.3% of the variation in the data. At the smallest scale, i.e. sites, only the environmental model was significant and explained 9.0% of the data variation after spatial effects were removed (Table 2C left). At this scale, the spatial model showed no significant patterns. The variation explained by spatially structured environmental variables [b] showed negative values.

Almost 10% of the variation in composition (using species abundance) at the scale of mainland-island was attributed to the environmental model, which included altitude, green cover and land slope (Table 2A center). After spatial effects were removed, the environmental model explained 10.0% of the variation in the data. The spatial model was also significant, but explained only 1.4%. Both models were significant when only the pure effects were analyzed. Spatially structured environmental variables [b] showed negative values.

At the area scale, the spatial effect was significant (explaining 17.4% of the variation) and greater than the environmental effect. Both models were significant when only the pure effects were analyzed, in which the spatial model explained 14.8% and the environmental model explained 7.3% of the variation (Table 2B center). The variation explained by spatially structured environmental variables [b] explained 2.5% of the variation in the data. At the site scale, the environmental model had greatest relative importance for dung beetle abundance (Table 2C center). After spatial effects were removed, the variables that composed the environmental model explained 15.8% of the variation in abundance data. The spatial model showed no significant spatial patterns at this scale. Spatially structured environmental variables [b] showed negative values.

At the mainland-island scale, analysis of the variation in species composition based on biomass showed that both environmental and spatial effects were significant (Table 2A right). The environmental model composed of altitude, green cover and land slope explained 9.9% of the variation, and spatial effects explained 2.8% of the variation in the data (after corrections). The variation explained by spatially structured environmental variables [b] explained 0.002% of the variation in the data.

At the area scale, the spatial model was significant and explained the greatest amount of the variation in the biomass data (16.6%) followed by the environmental model (9.9%) (Table 2B right). After correction, the spatial and environmental models explained 15.1% and 8.4%, respectively, of the variation in the biomass data. Spatially structured environmental variables [b] explained 1.5%. At the local scale, the environmental model explained 9.9% of variation, and when spatial effects were removed the proportion increased to 17.2%. The spatial model showed no significance at this scale. Values for the variation explained by spatially structured environmental variables [b] were negative.

## Discussion

In recent decades, there has been increased interest in understanding scale-dependence of the structuring processes of biological communities, including studies of protozoa [62], zooplankton [25], [63], ichthyoplankton [64], dragonflies [65], coral reefs [66], reef-fishes [67], freshwater fishes [65], plants [68], [69], frogs [65], birds [70], and mammals [71], covering a wide variety of ecosystems. The unique biology of dung beetles makes them excellent models with which to explore general concepts in ecology [31], including new approaches suggested by metacommunity theory. Our results represent a first step towards a better understanding of the relative importance of ecological processes on dung beetle community structure in a coastal mainland-island landscape across three different spatial scales.

In this study, the environmental heterogeneity had greater importance at smaller scales, and may be the cause of high beta diversity in terms of species richness and abundance (i.e., Shannon entropy) found among sampling sites. Local environmental factors seem to be crucial in the structuring of local communities; such factors may be responsible for high beta diversity at the local scale, as has been demonstrated for several groups of organisms in a variety of ecosystems [25], [72]–[77]. Thus, the ecological gradient evaluated here appears to have a distribution defined by spatially structured environmental heterogeneity, which may have strong effects on dung beetle community structure locally.

Beta diversity at the area scale was lower than at the site scale, despite the increase in geographic distance among the sampling points. At area scale, we found a significantly greater importance of spatial effects compared to environmental effects, even after the analysis of individual effects of the models. Beta diversity among areas appears to be mainly related to the spatial patterns of the sampling sites. The occurrence of shared environmental and spatial effects as drivers of beta diversity are very common with increasing spatial scale [24], [75], and these shared effects may suggest significant limitations in species dispersal ability between site and area scales. Besides environmental effects, spatial limitation may be related to geographic distance, lack of connectivity caused by fragmentation, or the landscape structure between the mainland and the island.

Between the mainland and the island, beta diversity showed the highest values and at this scale only the environmental model was significant for species composition, while for abundance the environmental and spatial models were significant. The high beta diversity found between the mainland and the island has its origin at site and area scales, where environmental and spatial patterns have high relative importance. Thus, we observed that the distribution of dung beetles along an ecological gradient occurs in a spatially structured environment, where such patterns may be generated due to dispersal limitation at intermediate scales, and due to environmental heterogeneity at local scales.

The distribution pattern of dung beetle species composition was associated with the altitude gradient. This variable was significant at all scales studied after spatial effects were removed, demonstrating its strong influence on the species composition of dung beetle communities. Altitude ranged between 28 and 265 m among sampling sites. A study performed in the Colombian Andes demonstrated that dung beetle composition varied along an altitude gradient between 1,000 and 2,250 m at intervals of roughly 250 m [78]; the differences found in this study were associated with different environmental adaptations of the species. Environmental and climatic differences are also important for dung beetle distribution at low altitudes. The proximity to the sea and the effect of wind on humidity [46], and soils with higher salt concentration, although not measured in our study, could also affect the relative success of some species. Thus, the environmental and spatial configuration of sampling sites evaluated in the mainland-island landscape may influence the distribution of dung beetle species.

Except for at the mainland-island scale, in general species composition and abundance showed similar responses to the ecological gradient studied. However, the relative importance of the models was greater for abundance data. Although abundance may not sufficiently explain patterns of species distribution (i.e., due to confounding effects caused by highly abundant species), it may help to explain the responses of species across the environmental gradients, because it reflects changes in the relative success of each species against these gradients [25]. In our study, abundance and biomass data were explained by the same set of environmental variables, and showed very similar responses to the ecological gradients. In general, dung beetle biomass was more influenced by individual spatial effects than abundance data. Thus, biomass may be an important descriptor of changes in the relative success of dung beetles along ecological gradients, because it is mainly derived from nutrients obtained from mammal feces [79], and availability of this resource may also be affected by environmental heterogeneity.

In addition to altitude, the percentage of green cover and land slope were part of the environmental model describing the distribution of dung beetle abundance and biomass. Green cover has also been found to explain the distribution of dung beetles species in different-sized Atlantic forest fragments [80]. Variation in the percentage of green cover illustrates the differences among sites with greater or fewer small plants and shrubs covering the soil. Sites with greater spacing between trees and less tree cover allow more sunlight, which may influence the microclimate and soil moisture, as shown to occur in forest edges [81]. Land slope ranged between five and 36° degrees, and having some degree of slope is a common characteristic among our sampling sites, due to the fact that Atlantic Forest is typically located on hillsides with a large altitudinal range [29]. In another study of Atlantic Forest in Serra do Japi, located in the western region of São Paulo state’s Atlantic plateau, Brazil, dung beetle composition was shown to vary between the tops, hillsides and valleys, which are associated with differences in environmental structure [46]. Sites with high degrees of land slope may be most affected by rainfall, and may present unfavorable soil features for some dung beetle species. These environmental characteristics may influence the distribution of dung beetles, and may have greater power to affect relative species success.

Changes in the structural complexity of forested areas may modify the entire community associated with these habitats, diminishing the species richness of some taxonomic groups and increasing the others [33]. For example, the structure of the environment was more important in determining dung beetle community composition than resource availability in areas occupied by cattle in Mexico [82]. The distribution of dung beetles along different environmental characteristics may show discrete associations typical to particular biotypes within the landscape [83]. Species richness, abundance and biomass of dung beetles were negatively affected in disturbed habitats (e.g., secondary forests and *Eucalyptus* plantations in the Brazilian Amazon) when compared to primary forest habitats [35]. Microclimatic variations in tropical forests related to canopy height and opening affected dung beetle communities in French Guiana [84]. Thus, many species of dung beetles have relationships with certain habitat characteristics, likely to facilitate finding mates and/or food, or could be directly related to the presence of organisms that produce their food resource.

High inter- and intraspecific competition, random distribution, and ephemeral nature of food resources together suggest, a priori, that dung beetles are probably good dispersers [85]. However, relatively few quantitative descriptions of dispersal in these beetles exist [85]. The dispersal ability of *Canthon acutus* was investigated in Venezuela using capture-mark-recapture technique [45]. The authors installed pitfall traps baited with feces at different distances in a semi-deciduous tropical forest, and they found that 95% of individuals were collected up to 25 m. In contrast, using similar techniques, other authors [86] evaluated the dispersal ability of *Canthon cyanellus cyanellus* across a Mexican landscape that contained different components such as forest fragments, hedgerows and pastures. They found a maximum movement distance among the different landscape components of 1,560 m for males (average 390 m) and 860 m for females (average 290 m), suggesting that landscape type change is not a barrier to dispersal for some species. In fact, some species from continuous Amazonian forest fragments do not extend their activities to adjacent open areas, and this effect is reduced when there is presence of secondary forest in these areas [40]. We may expect a similar pattern in the Brazilian Atlantic Forest.

Besides differences in dispersal ability, several species of dung beetles are associated with certain habitat types [35], [46], [47], [87]–[91] due to microclimatic factors [84] or resource availability [31]. Data on differences in dispersal ability in the species sampled in this study are still lacking. Based on our results, it is possible that the high beta diversity found among sites reflects low dispersal ability due to environmental and spatial effects. Many species of dung beetles that inhabit forests tend to not extend their range to open areas [40], [92], which influences their ability to disperse and colonize new habitats when the matrix is not favorable. However, species that live in forest edges or in the matrix [83], [86] may have a stronger ability to disperse and colonize new habitats compared to those living inside the forests.

The high beta diversity of dung beetle communities found among our sampling sites throughout the ecological gradient could still be related to historical events [5], [11] or neutral processes [10]. In a biogeographical context, the island of Santa Catarina shows similar physiographic and structural features to those of the mainland, since the island and the mainland were connected during past periods when the Atlantic Ocean level was low [93]. During that time, the small minimum distance between mainland and island (minimum of 500 m) and low maximum depth between them (about 30 m) may have allowed a favorable environment for dispersal of the species from the mainland to the island. Site “C” of ITA is unique in that is separated from the continuous forest that occurs in each area due to fragmentation caused by a highway; it had the lowest values for species richness (five) and for number of individuals (49), and was the exclusive site of occurrence of *Coprophanaeus dardanus*. This site also showed the lowest average altitude, and like others, this site has significant contribution to the negative relationship between LCBD values and community descriptors (species richness, abundance and biomass).

Due to the current fragmented structure of the landscape and the negative impacts on many coastal environments that urbanization has caused [93], [94], the Atlantic Forest landscape is highly fragmented and in different stages of succession, with each functioning as “islands”. The communities are isolated and dispersal and colonization rates are low [95] due to insertion in a matrix of inhospitable environments [96] for forest-inhabiting dung beetle species. Our results show that there were five species found only on the mainland, and five only found on the island. On the mainland, three species occurred only in ITA, and two only in ANH. On the island, two species occurred only in RAT, and two others were shared between RAT and PER. These results, as well as the analysis of the local contribution to beta diversity (significant sampling points occurred only near the ends of the spatial gradient, Figure 3) reflect the high importance of large-scale spatial effects in structuring dung beetle communities. The distinct occurrence of species between mainland-island may be result of isolation processes [95], [97], or local extinction due to lack of certain food sources (e.g., presence of certain mammals [38]) or simple inability to colonize [98]. Alternatively, species may persist at a given location due to biotic and/or abiotic conditions in spatially structured environmental conditions [99]. We propose that the processes listed above act as joint drivers of the current distribution of dung beetle species in the landscape studied, and our result suggest that the relative importance of each process depends on the spatial scale.

Environmental control (i.e., the species sorting paradigm) seems to be the dominant structuring process in the metacommunity at the local scale. However, environment was also important at larger scales, and environmental factors were spatially structured along the ecological gradient studied. Spatial effects were more important at larger scales, where there was an increase in beta diversity that appears to be due to limitation in dispersal ability of the species due to geographic barriers and fragmented landscape. Contrary to what was found in other studies [25], our results suggest that the increase in the spatial scale was related to increased environmental heterogeneity, although only mildly, agreeing with the general findings for stream insect communities [26]. We believe that our results, extrapolated with caution, represent general patterns that serve as the basis for other organisms with similar characteristics and requirements.

Appropriate management of spatially heterogeneous ecosystems requires an understanding of both local and regional processes by which beta diversity is created and maintained, in order to preserve the spatial organization or species-environment relationships on which beta diversity is dependent [12], [100]. Due to current scenario of fragmentation and isolation of remaining fragments of the Brazilian Atlantic Forest [29], knowing these answers is of great interest to managers and decision makers to plan appropriate conservation strategies in an increasingly human-modified world.

## Supporting Information

Figure S1 **Map of the sampling sites showing the distribution of species richness, abundance and total biomass of dung beetles.** ANH: Environmental Protection Area of Anhatomirim; ITA: Permanent Protection Area of Itapema; PER: Lagoa do Peri Municipal Park; RAT: Permanent Protection Area of Ratones. The circles represent the proportion to the total for each analysis.
(EPS)

Table S1 **Dung beetle species sampled in Atlantic Forest in eastern Santa Catarina, Brazil.** ANH: Environmental Protection Area of Anhatomirim in Governador Celso Ramos; ITA: Permanent Protection Area of Itapema; PER: Lagoa do Peri Municipal Park, Florianópolis; RAT: Permanent Protection Area of Ratones, Florianópolis. Letters A to E depict the sampled sites in each area. T: total.
(XLSX)

Dataset S1 **Dataset of abundance and dry biomass of dung beetle species, environmental variables, and geographical coordinates.** Samplings were performed in Brazilian Atlantic Forest, Santa Catarina, Brazil using baited pitfall traps from January to February 2012.
(XLSX)
